# Dual depletion of myeloid-derived suppressor cells and tumor cells with self-assembled gemcitabine-celecoxib nano-twin drug for cancer chemoimmunotherapy

**DOI:** 10.1186/s12951-024-02598-y

**Published:** 2024-06-08

**Authors:** Xiaojie Zhang, Qiangwei Liang, Yongjin Cao, Ting Yang, Min An, Zihan Liu, Jiayu Yang, Yanhua Liu

**Affiliations:** 1 Department of Pharmaceutics, School of Pharmacy, Ningxia Medical University, Yinchuan, 750004 China; 2 NHC Key Laboratory of Metabolic Cardiovascular Diseases Research, Ningxia Medical University, Yinchuan, 750004 China; 3 Department of Pharmacy, School of Nursing, Wuxi Taihu University, Wuxi, 214064 China

**Keywords:** Gemcitabine-celecoxib, Nano-twin drug, Myeloid-derived suppressor cells, COX-2/PGE_2_ pathway, Chemoimmunotherapy, Breast cancer

## Abstract

Myeloid-derived suppressor cells (MDSCs) have played a significant role in facilitating tumor immune escape and inducing an immunosuppressive tumor microenvironment. Eliminating MDSCs and tumor cells remains a major challenge in cancer immunotherapy. A novel approach has been developed using gemcitabine-celecoxib twin drug-based nano-assembled carrier-free nanoparticles (GEM-CXB NPs) for dual depletion of MDSCs and tumor cells in breast cancer chemoimmunotherapy. The GEM-CXB NPs exhibit prolonged blood circulation, leading to the preferential accumulation and co-release of GEM and CXB in tumors. This promotes synergistic chemotherapeutic activity by the proliferation inhibition and apoptosis induction against 4T1 tumor cells. In addition, it enhances tumor immunogenicity by immunogenic cell death induction and MDSC-induced immunosuppression alleviation through the depletion of MDSCs. These mechanisms synergistically activate the antitumor immune function of cytotoxic T cells and natural killer cells, inhibit the proliferation of regulatory T cells, and promote the M2 to M1 phenotype repolarization of tumor-associated macrophages, considerably enhancing the overall antitumor and anti-metastasis efficacy in BALB/c mice bearing 4T1 tumors. The simplified engineering of GEM-CXB NPs, with their dual depletion strategy targeting immunosuppressive cells and tumor cells, represents an advanced concept in cancer chemoimmunotherapy.

**Graphical Abstract:**

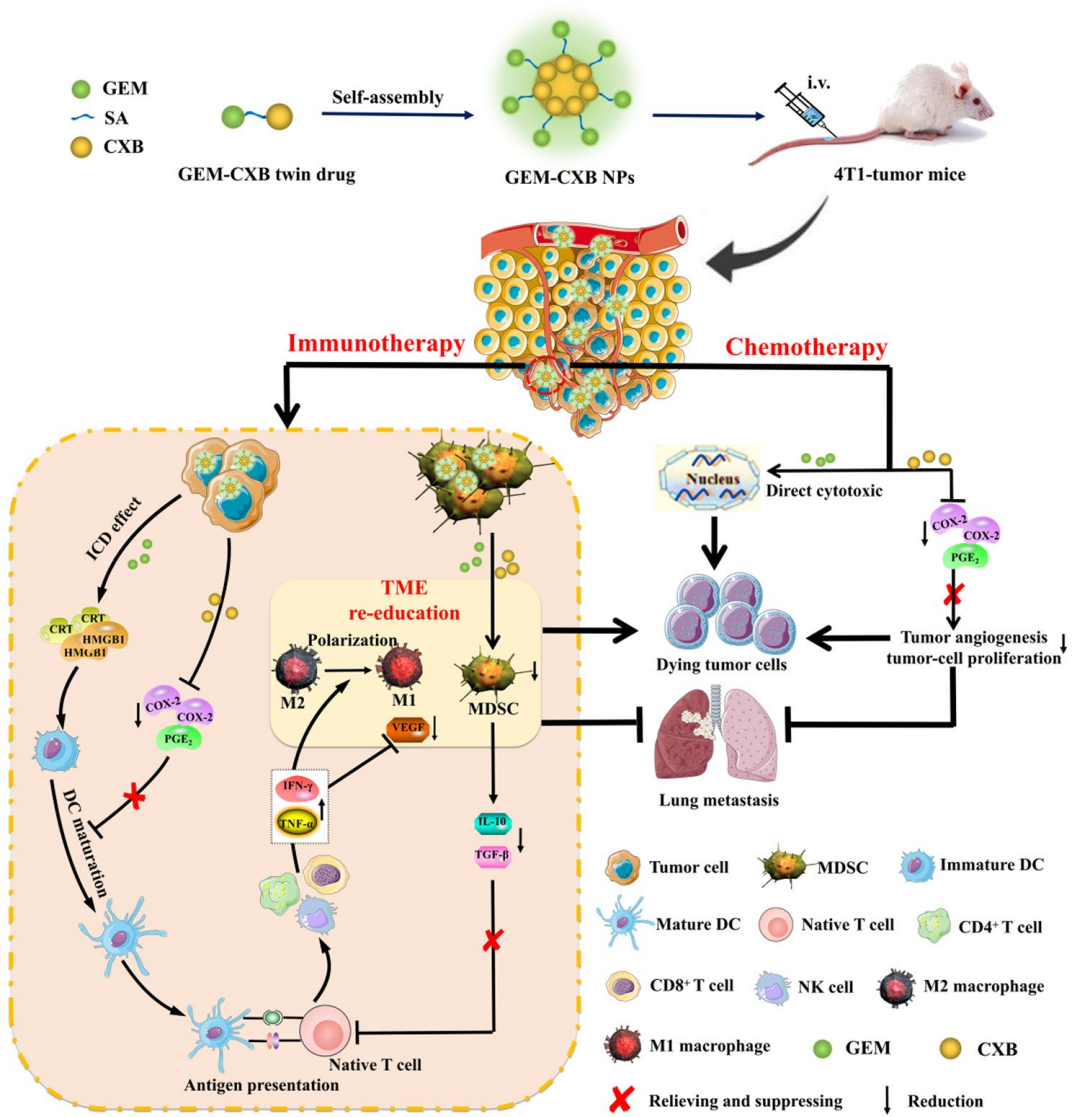

**Supplementary Information:**

The online version contains supplementary material available at 10.1186/s12951-024-02598-y.

## Introduction

Immunosuppression within the tumor microenvironment (TME) play a crucial part in promoting the growth, invasion and metastasis of tumors [1–4]. Although some chemotherapeutic agents can induce immunogenic cell death (ICD) in addition to their direct cytotoxic effects to tumor cells, the resulting innate immune response is usually very weak owing to the strongly immunosuppressive tumor microenvironment (ITM). Therefore, chemoimmunotherapy based on remodeling the ITM and improving the sensitivity of chemotherapy is recognized as a promising approach for cancer therapy [5, 6].

Myeloid-derived suppressor cells (MDSCs) are one of the key immunosuppressive cells in the TME, which play an indispensable role in inducing tumor immune escape and promoting immune tolerance. The MDSC-mediated ITM exerts the following effects to counter antitumor immune responses [7–9]: First, MDSC-induced immunosuppression hinders the maturation and recruitment of dendritic cells (DCs) through competing with antigen-presenting cells (APCs) for extracellular cystine uptake or through directly downregulating L-selectin levels on unsensitized T cells, which induces immune tolerance through inhibiting the activation and function of T cells. Second, MDSCs impair the cytotoxic function of natural killer cells (NKs) via overexpressing the transforming growth factor-β (TGF-β) and interleukin-10 (IL-10) or by reducing the secretion of interferon-γ (IFN-γ) [10–13]. Third, MDSCs promote the polarization of M2-like tumor-associated macrophages (TAMs) and recruit immunosuppressive regulatory T cells (Tregs). In addition, MDSCs induce tumor angiogenesis and metastasis via upregulating the expression of arginase 1, TGF-β, IL-10, and vascular endothelial growth factor (VEGF) [14, 15]. Therefore, the elimination of immunosuppressive MDSCs in the TME through signaling pathway regulations has become a prominent focus of cancer immunotherapy [16, 17].

The cyclooxygenase 2 (COX-2) overexpressed in tumors and its proinflammatory metabolite prostaglandin E2 (PGE_2_) not only blocks the migration of mature DCs but also recruits immunosuppressive MDSCs to increase the immunosuppressive barrier between tumor cells and T cells. Furthermore, the COX-2/PGE_2_ pathway is widely recognized for actively promoting tumor angiogenesis, stimulating tumor-cell proliferation, and protecting tumor cells from undergoing apoptosis [18–21]. Celecoxib (CXB), a nonsteroidal anti-inflammatory drug, exerts a chemotherapeutic effect by inhibiting the proliferation and migration of tumor cells, as well as tumor angiogenesis, through targeted inhibition of the COX-2/PGE_2_ pathway [22, 23]. Recent studies have also reported that CXB possesses immunoregulatory effects by improving the ITM through the direct depletion of MDSCs, and also through the COX-2/PGE_2_ pathway to alleviate inflammation-related immunosuppression [24]. This involves increasing the recruitment of DCs and infiltration of T cells, while reducing the infiltration of immunosuppressive cells [20, 25].

The chemotherapeutic drug gemcitabine (GEM), a cytidine nucleoside analog, exerts direct cytotoxic effects on tumor cells during chemotherapy. In addition, it triggers ICD, followed by stimulation of DCs to present tumor-associated antigens (TAAs) to T cells for activating antitumor immune response [26–28]. Furthermore, GEM has been proven to effectively deplete MDSCs in tumor-bearing mice [29–32]. However, the strong ITM poses a significant barrier, limiting the induction of ICD and depletion of MDSCs by GEM alone. Hence, we proposed a combination therapy involving the COX-2 inhibitor CXB and the ICD-inducer GEM for dual depletion of MDSCs and tumor cells for enhancing tumor cell immunogenicity. This approach promotes the recruitment and maturation of DCs, leading to the activation of cytotoxic T cells (CTLs) and NKs mediated antitumor immune responses. In addition, it suppresses the proliferation of Tregs and promotes the TAMs re-polarization from the M2 towards M1 phenotype, thus comprehensively regulating the antitumor immune functions of the human body. Furthermore, the combination of GEM and CXB synergistically enhances the chemotherapeutic activity, with CXB strengthening the antitumor effect of GEM-based chemotherapy. Overall, our study focuses on the combinatory therapy strategy involving GEM and CXB for dual depletion of MDSCs and tumor cells in breast cancer chemoimmunotherapy.

**Fig. 1:**
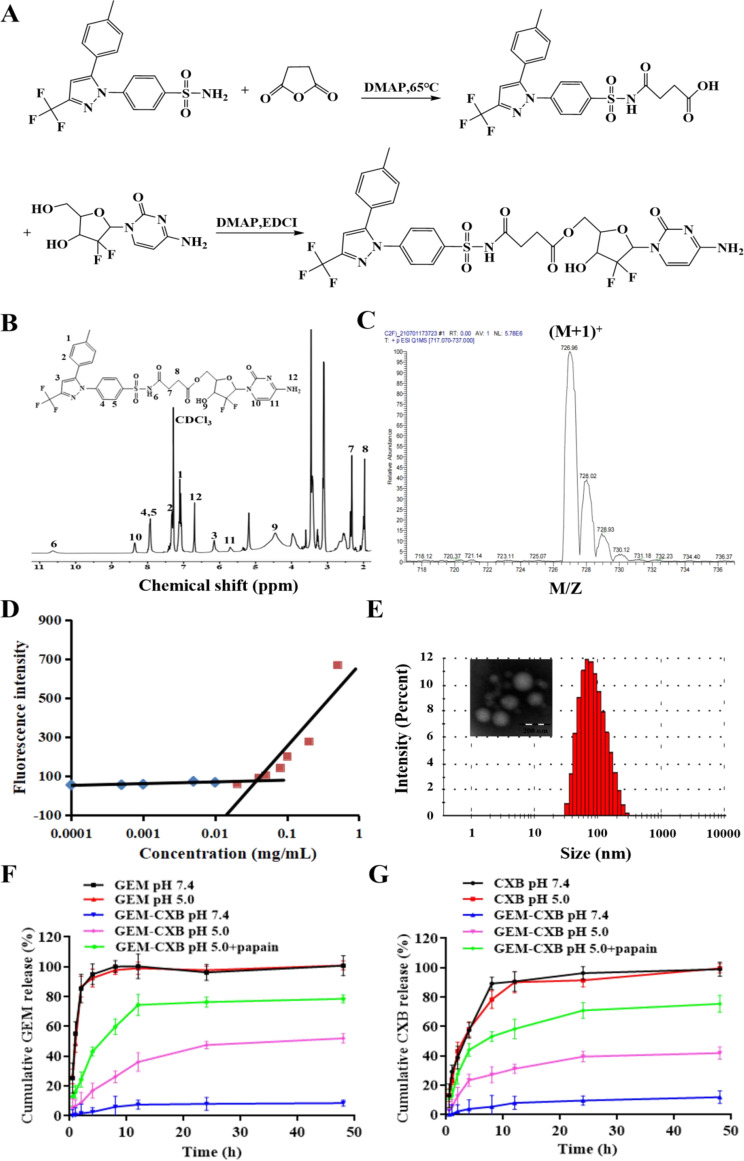
Schematic diagram of self-assembled GEM-CXB nano-twin drug for MDSCs and tumor cells dual depletion on breast cancer chemoimmunotherapy

However, the achievement of co-delivery of hydrophilic GEM and hydrophobic CXB in vivo to achieve a synergistic chemoimmunotherapy efficacy presents a considerable challenge. In recent years, carrier-free nano-prodrugs derived from small-molecule twin drugs have attracted increasing attention [30, 33]. In this study, an amphiphilic GEM-CXB twin drug was designed by conjugating hydrophilic GEM with hydrophobic CXB using a succinic acid linker. This design enabled the self-assembly of the twin drug into carrier-free nanoparticles, abbreviated as GEM-CXB NPs. As illustrated in Fig. 1, upon co-delivered into tumors, the GEM-CXB NPs exhibited several therapeutic effects: (I) The GEM-CXB NPs released GEM, contributing to the depletion of the tumor cells through its chemotherapeutic activity. In addition, the co-released CXB enhanced the chemotherapeutic actions on inhibiting the proliferation and inducing the apoptosis of tumor cells. (II) The GEM-CXB NPs induced ICD on releasing GEM, contributing to an antitumor immune response. The co-released CXB and GEM caused the dual depletion of MDSCs, further enhancing the antitumor immune activity. Based on the above chemoimmunotherapeutic actions, the GEM-CXB NPs showed promise in achieving an improved antitumor and anti-metastasis efficacy in 4T1 tumors.

To the best of our knowledge, the developed GEM-CXB small-molecule twin drug-based nanomedicine, which combines prodrug strategy and nanotechnology into a single system, along with its mechanism of dual depletion of MDSCs and tumor cells, represents an advanced platform for cancer chemoimmunotherapy.

## Materials and methods

### Materials

Gemcitabine (GEM) was supplied by Jiuding Chemical Technology Co., Ltd (Shanghai, China). Celecoxib (CXB), succinic anhydride (SA) and nile red (NR) were purchased from Aladdin Biochemical Technology Co., Ltd (Shanghai, China). Interleukin-2 (IL-2) was bought from MedChemexpress Biotechnology Company (NJ, USA). Cyclooxygenase-2 (COX-2), calreticulin (CRT) and high mobility group box 1 (HMGB1) antibodies were obtained from Biolegend Biological Co., Ltd (CA, USA). Carboxylfluorescein diacetate succinimide ester (CFSE) was supplied by Beibo Biotechnology Co., Ltd (Shanghai, China).

### Synthesis and characterization of GEM-CXB twin drug

CXB (1.0 equi), SA (1.0 equi) and DMAP (0.1 equi) were dissolved in ultra-dry DMF and reacted at 65 °C for 18 h under N_2_ atmosphere protection. The reaction solution was precipitated into cold hydrochloric acid (0.1 M) solution. The collected precipitates were then washed with ethyl acetate and saturated NaCl solution, respectively. Afterwards, the obtained crude product was purified with CH_2_Cl_2_/MeOH/CH_3_COOH (120/1/0.1, v/v/v) as the elution solvent by silica gel column chromatography, and the white powder CXB-SA was obtained after vacuum drying.

**Fig. 2:**
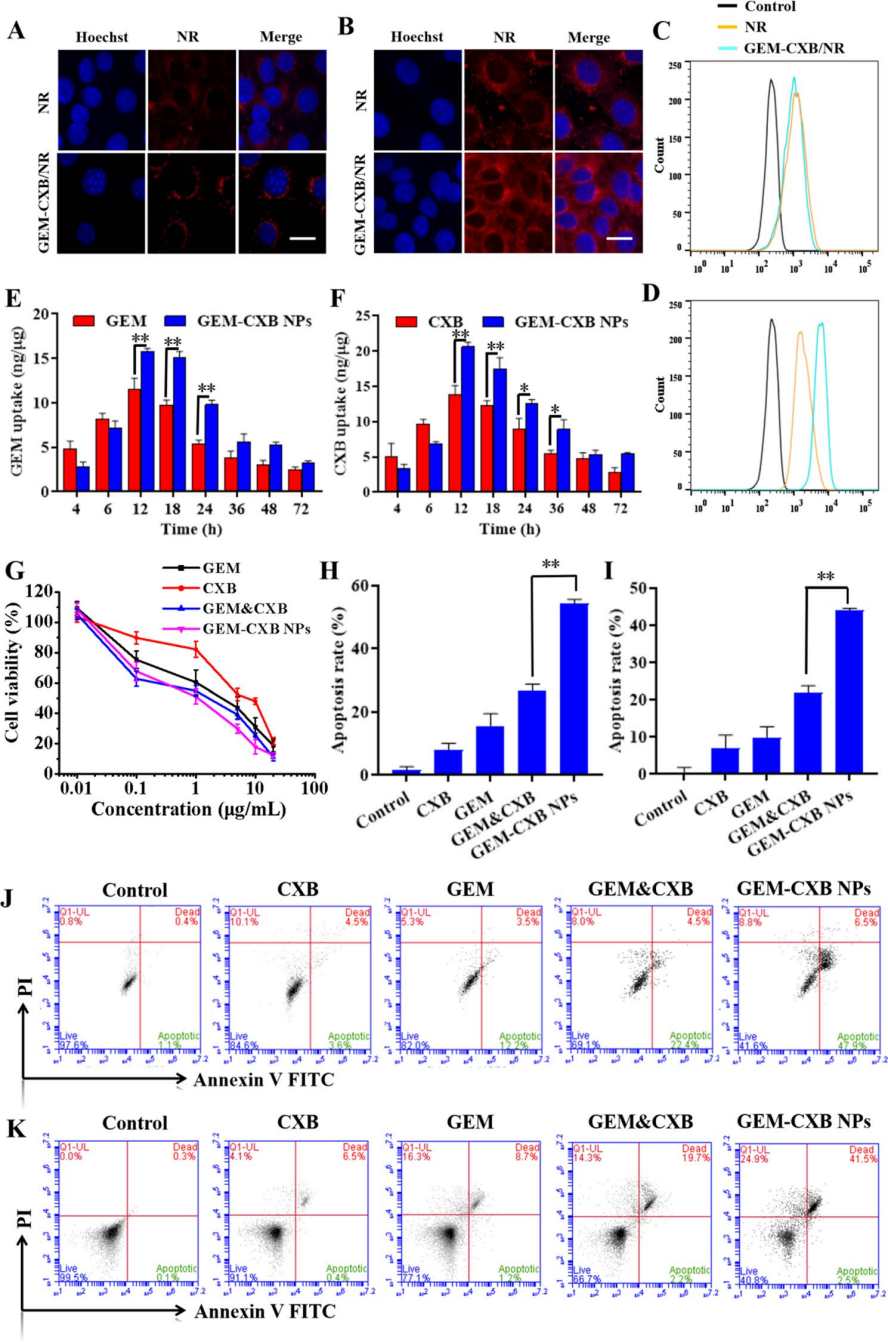
(**A**) Synthesis route of GEM-CXB twin drug. (**B**) ^1^H NMR spectra of GEM-CXB in CDCl_3_ and (**C**) ESI-MS spectrum of GEM-CXB. (**D**) Plots of NR fluorescence intensity vs. GEM-CXB concentrations. (**E**) The diagram of size distribution and TEM image of GEM-CXB NPs. Cumulative release profiles of GEM (**F**) and CXB (**G**) from GEM-CXB NPs at pH 7.4, pH 5.0, and pH 5.0 with 2.0 µM papain, with GEM and CXB solutions as controls

CXB-SA (1.0 equi), GEM (1.3 equi), EDCI (1.3 equi) and DMAP (0.1 equi) were dissolved in ultra-dry DMF and stirred for 24 h at room temperature. The reaction mixture was extracted and washed by ethyl acetate and hydrochloric acid (0.5 M) solution. The final product GEM-CXB was obtained by purification of the crude product with CH_2_Cl_2_/MeOH (30/1, v/v) as the elution solvent by silica gel column chromatography. The synthesis route of GEM-CXB twin drug is shown in Fig. 2A.

The synthesized GEM-CXB was identified by mass spectrometry (TSQ Access MAX, Thermo Fisher, USA) and ^1^H NMR (AVANCE III 400, Bruker, Switzerland), respectively. The critical micelle concentration (CMC) of the GEM-CXB was measured by the reported methods with nile red (NR) as fluorescence probe [34].

### Preparation and characterization of GEM-CXB twin drug NPs

GEM-CXB twin drug nanoparticles (abbreviated as GEM-CXB NPs) were prepared by solvent evaporation method [30]. The GEM-CXB twin drug was dissolved in 100 µL of methanol, slowly added dropwise to ultrapure water, and stirred overnight to obtain GEM-CXB NPs with light blue opalescence. The morphology, particle size and zeta potential of GEM-CXB NPs were observed by transmission electron microscopy (TEM, Tecnai 275 G220, FEI, USA) and determined by Zetasizer (Nano-ZS90, Malvern instruments), respectively.

The GEM-CXB NPs at gradient concentrations were incubated with the prepared 2% (v/v) red blood cell suspension at 37 °C for 8 h, with the PBS and Triton X-100 as negative and positive controls, respectively. The supernatant of each sample was measured at 540 nm by Microplate Reader, and the hemolysis rate of GEM-CXB NPs was calculated as follows:

$$\eqalign{{\rm{Hemolysis }}\left( {\rm{\% }} \right){\rm{ = }} & {\rm{ }}\left[ {{{\left( {{\rm{Abs}}} \right)}_{{\rm{sample}}}}{\rm{ - }}{{\left( {{\rm{Abs}}} \right)}_{{\rm{PBS}}}}} \right]{\rm{ }} \cr & {\rm{/ }}\left[ {{{\left( {{\rm{Abs}}} \right)}_{{\rm{Triton X - 100}}}}{\rm{ - }}{{\left( {{\rm{Abs}}} \right)}_{{\rm{PBS}}}}} \right]{\rm{ \times 100}} \cr}$$

The stability was performed by monitoring the particle size and PDI changes of GEM-CXB NPs incubated in PBS (pH 7.4) at 4 °C and PBS (pH 7.4, 10% FBS) at 37 °C, respectively, at designated time intervals. Moreover, the interaction between the GEM-CXB NPs and plasma or whole blood was investigated and represented by relative turbidity. In brief, GEM-CXB NPs at gradient concentrations were mixed with equal volume of plasma or whole blood in a 96-well plate, respectively, followed by incubated at 37 °C for designated time intervals.The absorbance of mixture were measured at 630 nm by Microplate Reader. The relative turbidity of each group was calculated by the ratio of absorbance at different time points to 0 h.

The drug release properties of GEM-CXB NPs were investigated with dynamic dialysis method in response to intracellular acidic and enzyme-rich endo/lysosomal environments. CXB, GEM and GEM-CXB NPs with CXB and GEM amounts of 300 µg and 200 µg, respectively, were transferred into dialysis bags (molecular weight cut-off of 3500 Da) and placed in PBS at pH 7.4 and pH 5.0, with and without papain (2.0 µM) at 100 rpm and 37 °C. The amounts of CXB and GEM released from GEM-CXB NPs at the designated time points were determined using previously reported HPLC methods [20, 30].

### Cells and animals

Mouse breast cancer 4T1 cells (the Cell Bank of the Chinese Academy of Sciences) were cultured in RPMI 1640 medium containing FBS (10%, v/v) with penicillin (100 IU/ml) and streptomycin (100 µg/ml) at 37 °C in a humidified 5% CO_2_-95% air atmosphere.

Female ICR and BALB/c mice (the Animal Center of Ningxia Medical University) aged 6–8 weeks were housed under pathogen-free conditions. The animal experiments were carried out according to the Guidelines for Laboratory Animals by the Ethics Committee of Animal Experimentation of Ningxia Medical University.

### Intracellular uptake and drug release

4T1 cells incubated in a 6-well culture plate (3 × 10^4^ cells/well) with coverslips were treated with NR-loaded GEM-CXB NPs, prepared according to the previous methods with NR addition, in comparison to NR as a control. The cells were stained with Hoechst 33342 after 2 h and 4 h treatments, fixed with 4% paraformaldehyde, followed by confocal laser scanning microscopy (CLSM, FluoView 1000, Olympus, Japan) observation. Moreover, the cells post 2 h and 4 h treatments were trypsinized, centrifuged, suspended in PBS (pH 7.4), and analyzed using flow cytometry (BD Biosciences, Franklin Lakes, NJ).

4T1 cells incubated in a 6-well culture plate (2 × 10^5^ cells/well) were treated with GEM-CXB NPs at different time intervals, with GEM and CXB as controls, followed by lysing by 0.1% Triton X-100. The intracellular GEM and CXB release from GEM-CXB NPs were determined by the established HPLC methods, and expressed as GEM and CXB amount per microgram protein assayed by the BCA protein assay kit.

### In vitro proliferation inhibition and apoptosis induction against 4T1 cells

The in vitro anti-tumor activity of GEM-CXB NPs was performed by investigating the cytotoxicity and apoptosis against 4T1 cells. 4T1 cells incubated in a 96-well culture plate (3 × 10^3^ cells/well) were treated with the CXB, GEM, GEM&CXB and GEM-CXB NPs at gradient GEM&CXB concentrations. Following 72 h incubation, the cell viabilities were measured with MTT assays [35]. The apoptotic rate of 4T1 cells following various formulations treatment for 24 h were stained by Annexin-FITC/PI Apoptosis Detection Kit and analyzed with flow cytometry (BD Biosciences, Franklin Lakes, NJ).

### In vitro COX-2 expression and PGE_2_ level analysis

4T1 cells incubated in a 6-well culture plate (5 × 10^4^ cells/well) were treated with the CXB, GEM, GEM&CXB and GEM-CXB NPs. After 48 h incubation, the cells were lysed, centrifuged, and the supernatants were quantified. Proteins with equal amounts were separated by 10% SDS-PAGE electrophoresis, followed by electrotransferring onto polyvinylidene difluoride membranes. After being blocked with 5% Difco skimmed milk, the proteins were incubated overnight at 4°C with primary antibodies against β-actin and COX-2. Afterwards, the membranes were incubated with a secondary antibody at room temperature for 1 h, followed by acquiring with the chemiluminescence system (Thermo Scientific) and quantifying by Image J software. Moreover, the PGE_2_ amounts in the supernatants were analyzed by ELISA kits (MultiSciences, LiankeBio).

### ICD induction and DCs maturation

The calreticulin (CRT) exposure and high mobility group protein B1 (HMGB1) releasing induced by GEM-CXB NPs were evaluated by immunofluorescence and western blot, respectively. 4T1 cells incubated in a 6-well culture plate (5 × 10^4^ cells/well) were treated with CXB, GEM, GEM&CXB and GEM-CXB NPs. Following 24 h incubation, the cells were treated with CRT rabbit monoclonal antibody and Alexa Fluor 488-goat anti-rabbit IgG, respectively, and further stained with Hoechst 33,342, followed by observation under an inverted fluorescence microscope. The cells were lysed, and the HMGB1 levels in the supernatants were assayed through western blot.

4T1 cells incubated in a 6-well culture plate (2 × 10^5^ cells/well) were treated with various formulations. The supernatants of the 4T1 cells post 24 h treatments were collected and co-incubated with the prepared bone marrow derived dendritic cells (BMDCs, 8 × 10^5^ cells/well) for 24 h. Then, the collected BMDCs were treated with APC anti-mouse CD11b, FITC anti-mouse CD40 and AlexaFluor 488 anti-mouse CCR7 antibodies for 30 min, followed by analyzing with flow cytometry. Cells with PBS treatment was used as control.

### In vitro MDSCs apoptosis and T cell proliferation

The BALB/c mice bearing 4T1 tumors established by s.c. inoculation of 4T1 cells (2 × 10^6^ cells/mouse) were sacrificed, the spleens were excised, and the single splenocyte suspensions were prepared and red blood cells were lysed. The cells were treated with the PE-Gr-1 and APC-CD11b antibodies, then the MDSCs were sorted and purified by flow cytometry. The purified MDSCs incubated in a 6-well culture plate (5 × 10^4^ cells/well) were treated with various formulations. After 24 h treatments, the MDSCs apoptosis induction was subsequently assayed using Annexin-FITC/PI Apoptosis Detection Kit.

Mouse splenocytes containing lymphocytes, DCs, monocytes and other cells were utilized to simulate in vivo immune microenvironment. The CFSE-labeled splenocytes collected from the spleen in BALB/c mice bearing 4T1 tumors were incubated with the purified MDSCs overnight, subsequently treated with various formulations, supplemented with IL-2 and CD3 antibodies incubation. Then the cells post 24 h treatments were treated with PE-CD4 and APC-CD8 antibodies for 30 min, afterwards analyzed the T lymphocytes proliferation with flow cytometry.

### In vivo pharmacokinetics and biodistribution

ICR mice were i.v. injected with GEM-CXB NPs with CXB and GEM as controls, at 7 mg GEM/kg and 10 mg CXB/kg dosages, respectively. Blood samples were collected at designated time points and centrifuged. The obtained plasma was mixed with internal standard (carbamazepine or 5-fluorouracil) and acetonitrile, vortexed and centrifuged. The plasma concentrations of CXB and GEM were determined using the established HPLC methods, and the pharmacokinetic parameters were analyzed via DAS 2.0 software.

For in vivo GEM and CXB distribution study, the BALB/c mice bearing 4T1 tumors grown to approximately 500 mm^3^ were i.v. injected with CXB, GEM and GEM-CXB NPs and sacrificed at 24 h post-injection. The tissues and tumor were excised, weighed, homogenized with saline, extracted and analyzed the CXB and GEM amounts in tissue homogenates according to the procedure as plasma samples done.

### In vivo anti-tumor and anti-lung metastasis

BALB/c mice bearing 4T1 tumors with volume of about 50 mm^3^ were i.v. injected with saline, CXB, GEM, GEM&CXB and GEM-CXB NPs at GEM and CXB doses of 7 mg/kg and 10 mg/kg, respectively, with 3-day intervals for 3 times injection. The tumor length (L) and width (W), including the body weight were measured every 3 days after treatments. The tumor volume was calculated following the equation of L × W^2^/2. The relative tumor volume was expressed as the ratio of the tumor volume measured on the day to the volume at the first treatment.

At the end of the in vivo anti-tumor activity investigation, the excised lungs were photographed and the number of lung metastatic nodules were recorded. The collected tumors and lungs of mice were performed by hematoxylin and eosin (H&E) analysis for pathological changes observation. Moreover, the apoptosis extents of tumor cells with different treatments was analyzed with terminal deoxynucleotidyl transferase dUTP nick-end labeling (TUNEL) kit. Furthermore, the survival time of the 4T1 tumor-bearing BALB/c mice (*n* = 8) with indicated treatments was monitored in a separate in vivo therapeutic efficacy study. The end point of survival was recorded when the mice were deceased or the tumor volume reached to about 1000 mm^3^.

### In vivo safety evaluation

At the end of the in vivo anti-tumor activity investigation, the levels of aspartate aminotransferase (AST) and alanine aminotransferase (ALT), blood urea nitrogen (BUN) and creatinine (Crea) in the collected serum, as the indicators of renal and hepatic functions, were analyzed. Whole blood was collected for whole blood cellular components counting. Moreover, the main organs of mice with the indicated treatments were performed by H&E analysis for the potential pathological observation.

### Chemoimmunotherapy mechanisms of GEM-CXB NPs

The excised tumors with the indicated treatments were lysed with enhanced RIPA lysis buffer, with the supplement of 1% phenylmethanesulfonyl fluoride. The tumor lysates were extracted and the amounts of HMGB1, COX-2, NF-κB and STAT3 were performed with western blot.

One day after the last treatment, the excised tumors following various treatments were digested by Liberase TL and DNase I for 0.5 h, subsequently ground and filtered through a 200-mesh strainer, centrifuged and washed twice with D-Hanks. The prepared single cell suspensions were treated with fluorescent-labeled antibodies and analyzed by flow cytometry. The cytokine levels of INF-γ, PGE_2_, VEGF, TGF-β, IL-10 and TNF-α in tumors were analyzed by ELISA.

The tumors isolated from 4T1 tumor-mice with the indicated treatments were frozen-sectioned, and then stained with fluorescent-labeled antibodies and visualized using CLSM for immunofluorescence analyzing the presence of CD4^+^CD8^+^ T cells, mature DCs and MDSCs, and for immunohistochemistry analyzing the COX-2 and HMGB1 levels in tumors.

### Statistical analysis

All data are expressed as mean ± standard deviation (SD). Statistical analysis was carried out with two-tailed Student’s t test between two groups and one-way analysis of variance (ANOVA) for multiple groups, followed by Newmane-Keuls test if *p* < 0.05. In all statistical analyses, *p < 0.05 was considered statistically significant, and **p < 0.01 was considered highly statistically significant.

## Results and discussion

### Preparation and characterization of GEM-CXB NPs

The amphiphilic GEM-CXB twin drug was synthesized by chemical conjugation of the hydrophilic chemotherapeutic agent GEM with the hydrophobic COX-2 inhibitor CXB using succinic anhydride as a linker. This method offered several advantages such as easy synthesis, precise structure, and a high and fixed drug loading capacity. The chemical structures of the GEM-CXB twin drug were identified by ^1^H NMR and ESI-MS (Figure S1A-B). As shown in Fig. 2B, the proton signals corresponding to the benzene ring located in CXB were observed at 6.5-8.0 ppm (signals 1, 2, 4 and 5). The characteristic peaks at 5.3 ppm (signal 6) and 1.5-3.0 ppm (signals 7 and 8) were belonged to the -NH- and -CH_2_- proton signals of succinic anhydride, respectively. Moreover, the peaks at 5.6 ppm, 8.4 ppm, and 6.63 ppm were attributed to the proton signals of -HC = CH- and -NH_2_ in the cytidine ring of GEM. In addition, a [M + H]^+^ ion spectra peaked at m/z of 726.95 was presented in the ESI-MS spectrum, which corresponded to the theoretical calculated molecular weight of GEM-CXB (m/z, [M + H]^+^, 727.1, Fig. 2C), which further confirmed the successful synthesis of the GEM-CXB twin drug. Furthermore, the GEM-CXB twin drug exhibited a lower critical micelle concentration of 45.0 µg/ml (Fig. 2D), indicating its self-aggregation performance in aqueous solutions and structural stability against dilution in the bloodstream after i.v. injection.

The GEM-CXB twin drug owing to its inherent amphiphilicity could self-assemble via the solvent evaporation method into well-defined and stable nanoparticles in an aqueous solution. As shown in Fig. 2E, GEM-CXB NPs exhibited an average particle size of 96.5 nm with a polydispersity index of 0.3, which was consistent with the uniform spherical particles observed in TEM images. The zeta potential of the GEM-CXB NPs was 2.12 mV. The GEM-CXB NPs offer the advantages of both the twin drug and nanoparticles, with a fixed and high drug loading capacity. The determined CXB and GEM contents in GEM-CXB NPs were 52.5% and 36.2%, respectively. Less than 2% hemolysis ratios of GEM-CXB NPs was observed within 8 h, confirming their good blood compatibility as a safe nano-delivery system for i.v. injection (Figure S1C). Dynamic light scattering measurements at different time points showed that GEM-CXB NPs remained almost unchanged in pH 7.4 PBS over 7 days, and a slightly particle size increased of 30 nm during a period of 48 h incubation in pH 7.4 PBS containing 10% FBS (Figure S2). Moreover, as shown in Figure S3, with the increased in incubation time, the relative turbidity of the GEM-CXB NPs increased slightly compared with the saline group. GEM-CXB NPs had good stability both in plasma and whole blood, implying that GEM-CXB NPs would avoid serum or whole blood-induced aggregation and maintain good structural integrity during blood circulation, which exhibited prolonged circulation in blood, and preferential accumulation in tumors.

In a period of the first 2 h, free GEM and CXB displayed rapid release profiles, and complete release were observed after 8 h and 10 h in release medium at pH 7.4 and pH 5.0, respectively, which was detrimental to its blood circulation (Fig. 2F and G). On the contrary, the GEM-CXB NPs showed only about 8.5% of GEM and 5.0% of CXB cumulative release at pH 7.4, whereas a more than 40.0% and 38.0% of pH responsive-triggered GEM and CXB release were found at pH 5.0 after 48 h incubation. Notably, a dramatically accelerated and nearly 80% of the GEM and CXB release profiles were found from GEM-CXB NPs in a period of 48 h at pH 5.0 containing 2.0 µM papain. Taking the above results into account, it was further confirmed that GEM-CXB NPs could be beneficial to realizing minimal drug leaking during circulation in blood, which was favorable for long-acting co-delivery in the blood circulation and efficient tumor accumulation. The endo/lysosomal pH and enzyme-rich environment in tumor cells could trigger GEM&CXB efficient release behaviors upon endocytosed thereafter by 4T1 cells, thus exerting synergistic MDSCs and tumor cells dual depletion actions on cancer chemoimmunotherapy.

### Intracellular uptake and drug release

**Fig. 3:**
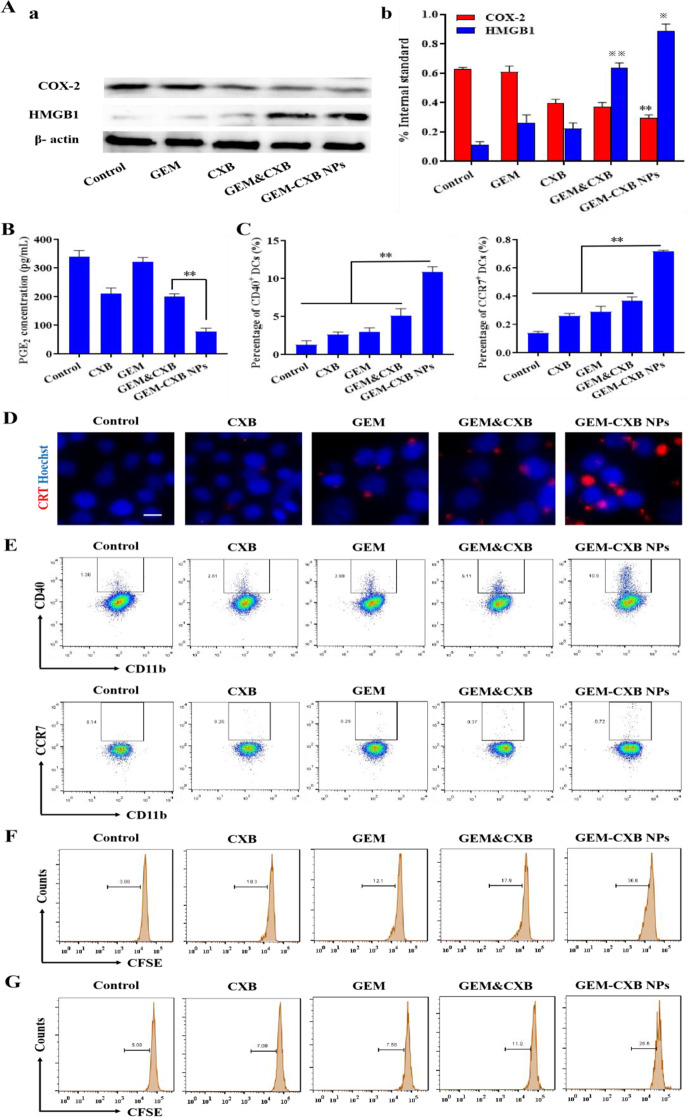
The NR internalization efficiency in 4T1 cells observed by CLSM and analyzed via flow cytometry with NR solution and GEM-CXB/NR NPs treatments for 2 h (**A**, **C**) and 4 h (**B**, **D**), the scale bar is set to 10 μm. Intracellular release of GEM (**E**) and CXB (**F**) at different time points after various formulations-treatment. (**G**) In vitro cytotoxicity profiles of 4T1 tumor cells against different formulations-treatment for 72 h. In vitro cell apoptosis induction and quantitative analysis of apoptosis rate in 4T1 cells (**H**, **J**) and MDSCs (**I**, **K**) against different treatments

**Fig. 4:**
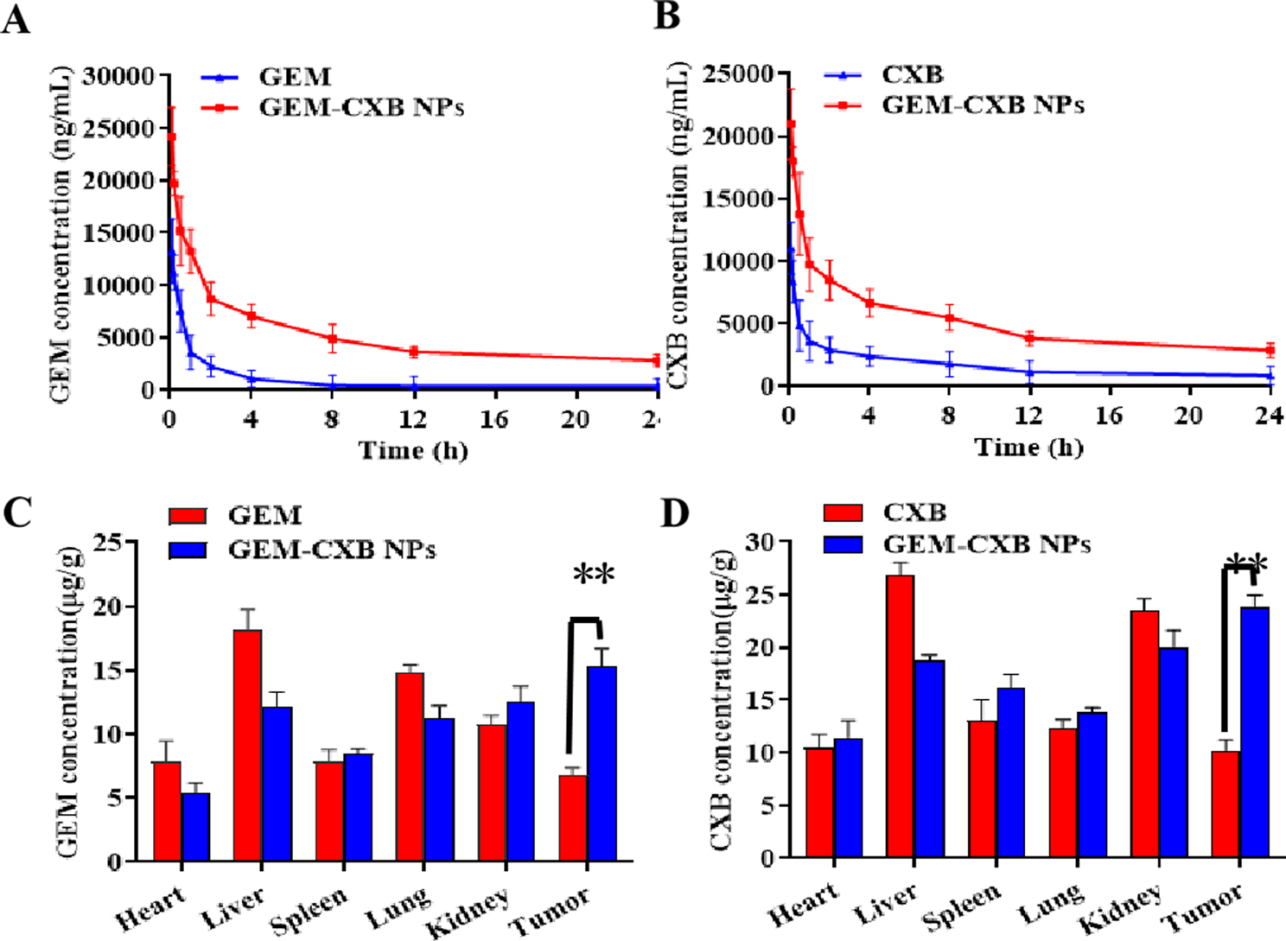
(**A**) HMGB1 and COX-2 protein expressions in 4T1 tumor cells analyzed by western blot (a) and quantified via Image J software (b) after treatment with different formulations. ^**^p < 0.01 (GEM-CXB NPs vs. Saline), ^※※^*p* < 0.01 (GEM&CXB vs. Saline or GEM or CXB), ^※^*p* < 0.05 (GEM-CXB NPs vs. GEM&CXB). (**B**) The secretion level of PGE_2_ in 4T1 tumor cells treated with different formulations analyzed by ELISA kit. (**C**, **E**) Quantitative analysis of the percentage of CD40 and CCR7 (**I**) after different treatments. (**D**) The CRT exposure on 4T1 tumor cells treated with different formulations observed by inverted fluorescence microscope. Flow cytometry analysis of CD4^+^ (**F**) and CD8^+^ (**G**) T cell proliferation after co-culture of MDSCs and CFSE-labeled splenocytes with different formulations-treatment

The internalization performance of GEM-CXB NPs into 4T1 cells was qualitatively and quantitatively investigated via CLSM and flow cytometry, respectively. As presented in Figs. 3A and B and 4T1 cells exhibited comparable amounts of fluorescent signals after being treated with free NR and GEM-CXB/NR NPs for 2 h. However, after 4 h of treatment, compared to 4T1 cells treated with NR alone, a considerably stronger NR fluorescence signal was observed in 4T1 cells with GEM-CXB/NR NPs treatment, which was consistent with the results of fluorescence quantitative analysis by flow cytometry (Fig. 3C and D). This might be attributable to the fact that GEM-CXB NPs with uniform spherical size were more favorable to be endocytosed by tumor cells and exhibited a time-dependent endocytosis pattern.

The effective release of drugs into cells is important for achieving synergistic antitumor effects with GEM-CXB NPs. As shown in Fig. 3E and F, more drugs were detected in cells treated with free GEM and CXB within the first 4 h compared to those treated with GEM-CXB NPs. This difference may be attributable to the rapid passive diffusion of free small-molecule drugs into cells, which can occur faster than the endocytosis pathway for nanoparticle internalization. However, considerably higher amounts of GEM and CXB were released from GEM-CXB NPs as the incubation time extended to 12 h. This may be attributable to hydrolysis and cleavage of the GEM-CXB twin drug in response to the intracellular pH and enzyme-rich endo-lysosomal environments after a large number of GEM-CXB NPs were endocytosed into cells. This can generate synergistic effects in depleting MDSCs and tumor cells, facilitating cancer chemoimmunotherapy.

### In vitro proliferation inhibition and apoptosis induction against 4T1 cells

The in vitro antitumor activity of GEM-CXB NPs was studied by assessing their synergistic effects on the proliferation inhibition and apoptosis induction against 4T1 cells. The 4T1 cells treated with free GEM, CXB, GEM&CXB combo, and GEM-CXB NPs showed concentration-dependent cell growth inhibition profiles. The GEM&CXB combo showed enhanced inhibition efficacy of tumor cell growth compared to the effects of individual drugs used alone (Fig. 3G), and promoted 26.9% of cell apoptosis compared with 8.1% and 15.7% of cell apoptosis induced by treatment with free CXB and GEM alone, respectively (Fig. 3H and J). These findings confirmed the synergistic antitumor effects of a combination of GEM and CXB. It is worth to notice that owing to the effective intracellular co-release of GEM and CXB after being efficiently endocytosed into cells, GEM-CXB NPs showed a synergistic action and exerted the most potent antitumor activity in 4T1 cells. They showed enhanced proliferation inhibition and induced 54.0% of cell apoptosis, surpassing the effects of the GEM&CXB combo.

### In vitro COX-2 expression and PGE_2_ secretion

The COX-2/PGE_2_ pathway promotes tumor immunosuppression by hindering the maturation of DCs and indirectly recruiting MDSCs. Therefore, the effects of indicated treatments on COX-2 expression and PGE_2_ secretion in 4T1 cells were investigated. As shown in Fig. 4A and B, GEM-treated cells had no effects on the expression of COX-2 and secretion of PGE_2_. By contrast, in comparison to the limited inhibition of COX-2 expression and PGE_2_ secretion shown by free CXB and GEM&CXB combo-treated cells, GEM-CXB NPs-treated 4T1 cells exhibited a more significant decrease in COX-2 expression and PGE_2_ secretion. This could be attributed to the greater amounts of CXB endocytosed and released inside the cells. The suppression of the COX-2/PGE_2_ pathway could be beneficial in inhibiting the intratumoral recruitment of MDSCs, promoting the maturation and recruitment of DCs, as well as strengthening the antitumor efficacy of GEM-based chemotherapy to synergistically improve the overall chemoimmunotherapy outcome.

### ICD induction and DCs maturation

As reported, GEM can induce ICD and further stimulate the DCs maturation, followed by activation of the antitumor immune activity. Therefore, the ICD-inducing ability of GEM-CXB NPs was evaluated by examining the membrane exposure of CRT and the expression of HMGB1 protein in 4T1 tumor cells. Figure 4A and D show that no obvious CRT fluorescence signal or HMGB1 expression was observed in CXB-treated cells. GEM&CXB combo-treated cells showed remarkably more CRT exposure and HMGB1 expression than those in GEM-treated cells. It could be explained that CXB relieved the inflammation-related immunosuppression through the COX-2/PGE_2_ pathway and it synergistically enhanced the chemotherapeutic activity of GEM for strengthening the ICD induction effect of GEM. Most important, GEM-CXB NPs-treated cells exhibited the most CRT exposure on their membranes and HMGB1 expression. The efficient induction of ICD by GEM-CXB NPs was conducive to enhance the immunogenicity of 4T1 cells and further stimulate the maturation of DCs, which would be beneficial in activating the antitumor immune response.

The maturation and migration of DCs are important to activate the antitumor immune response through capturing and presenting TAAs to T lymphocytes in the lymph nodes. The DCs maturation and migration abilities were investigated by assaying the expressions of CD11b^+^CD40^+^ DCs and CCR7^+^ DCs by co-incubation of different formulations treated-4T1 cells with BMDCs. As shown in Fig. 4C and E, the frequencies of CD11b^+^CD40^+^ DCs (5.1%) and CCR7^+^ DCs (0.37%) in the GEM&CXB combo were 1.7, 2.0 and 3.9-fold higher than those in cells with individual GEM and CXB treatment, as well as the untreated cells, respectively. This was achieved by the synergistic effects of GEM-induced ICD and CXB-medicated blockade of the COX-2/PGE_2_ pathway. Importantly, GEM-CXB NPs showed the highest capability to stimulate the maturation and migration of DCs, with frequencies of CD11b^+^CD40^+^ DCs and CCR7^+^ DCs reaching 10.9% and 0.72%, respectively. The improved maturation and migration of DCs might be attributed to the GEM&CXB combination strategy and the efficient intracellular co-delivery of GEM and CXB through the nano-twin drug approach, which ultimately increased tumor immunogenicity and induced the generation of antitumor immune activity.

### In vitro apoptosis of MDSCs and T cell proliferation

T cell function can be suppressed by MDSCs, resulting in immune escape, tumor progression, and metastasis in the ITM. It has been reported that GEM and CXB can selectively deplete MDSCs and alleviate their immunosuppressive effects on T cells. Therefore, the in vitro ability of GEM-CXB NPs to deplete MDSCs and promote T cell proliferation was analyzed via apoptosis assay and flow cytometry, respectively. As shown in Fig. 3I and K, treatment with GEM alone resulted in a 9.9% apoptosis rate in MDSCs, while CXB treatment alone resulted in a 6.9% apoptosis rate. GEM&CXB combo induced a higher rate of apoptosis of MDSCs of 21.0%, which further demonstrated the synergistic effect of GEM&CXB combo in depleting MDSCs. It was worth noting that treatment with GEM-CXB NPs led to a remarkable MDSC apoptosis rate of 44.0%, which was 2.1 folds higher compared to that induced by the GEM&CXB combo treatment. This action is expected to alleviate the immunosuppressive effects of MDSCs on the T cell proliferation and immune function.

The effect of GEM-CXB NPs on T cell proliferation and depletion of MDSCs was further evaluated. As shown in Fig. 4F and G, MDSCs incubated with untreated splenocytes resulted in low exhibiting proliferation with only 3.9% and 5.1% proportions of CD4^+^ and CD8^+^ T cells, respectively, indicating the immunosuppressive effect of MDSCs on T cell proliferation. After treatment with free CXB and GEM, the proliferation proportion of CD4^+^ and CD8^+^ T cells increased to 10.1%, 12.3% and 7.1%, 7.6%, respectively. The GEM&CXB combo treatment led to even higher proportions of CD4^+^ and CD8^+^ T cell proliferation, with 17.9% of CD4^+^ and 11.0% of CD8^+^ T cells proliferating, which suggested that the suppression of T cell proliferation was alleviated through the synergistic depletion of MDSCs by the GEM&CXB combo. Importantly, treatment with GEM-CXB NPs resulted in the highest activation of T cell proliferation, with the proportions of CD4^+^ and CD8^+^ T cells reaching 36.6% and 26.5%, respectively. This increased proliferation can be attributed to the preferential depletion of MDSCs by the GEM-CXB NPs.

In summary, GEM-CXB NPs brought about the depletion of MDSCs, induction of ICD, and maturation of DCs, highlighting their potential in enhancing T cell-triggered antitumor immune activity and improving the chemoimmunotherapy efficacy for 4T1 tumors.

### In vivo pharmacokinetics and biodistribution

**Fig. 5:**
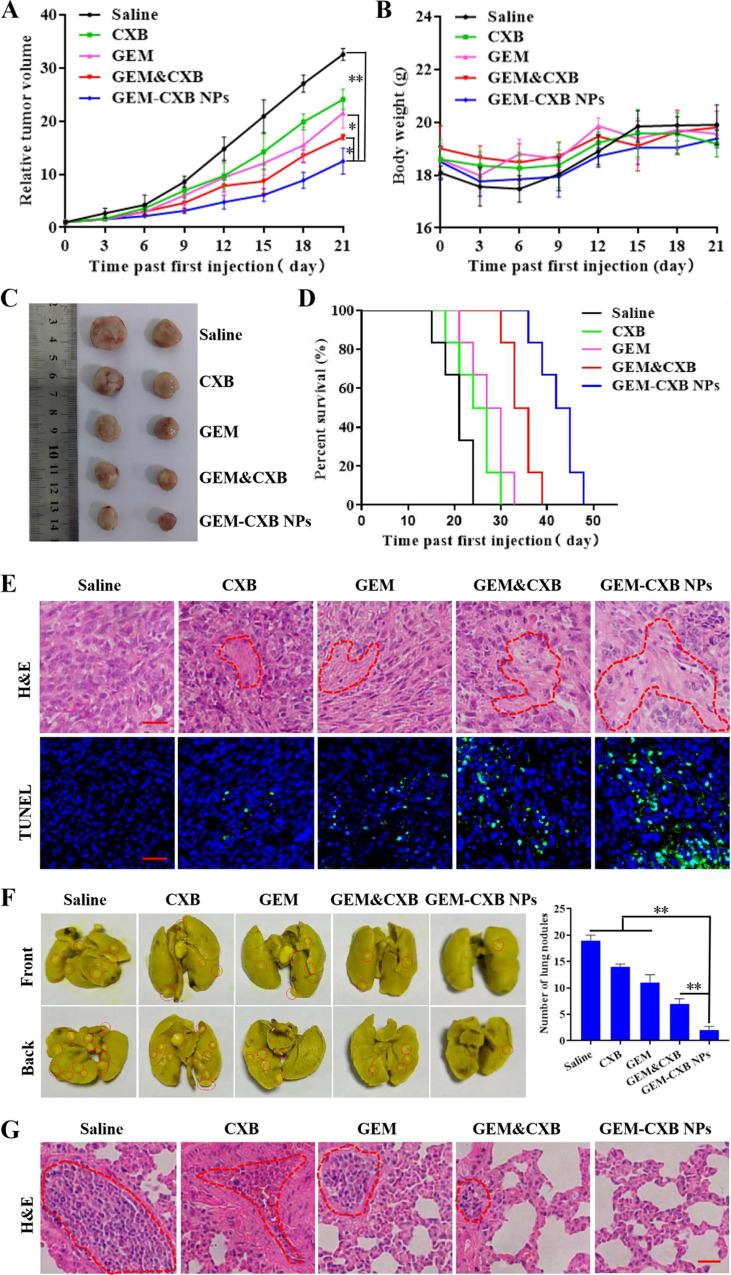
In vivo pharmacokinetics profiles of GEM (**A**) and CXB (**B**), and GEM (**C**) and CXB (**D**) biodistribution in 4T1-tumor mice after i.v. injection with different formulations

The pharmacokinetic profiles of CXB and GEM in mice treated with GEM-CXB NPs are shown in Fig. 5A and B. When GEM and CXB were intravenously administered in mice, they were rapidly eliminated from the bloodstream, leading to a sharp reduction in their plasma levels. By contrast, when GEM and CXB were administered as GEM-CXB NPs, the clearance of both drugs was considerably delayed. As listed in Table S1-S2, compared to mice treated with individual drugs, the half-life values (t_1/2β_) of GEM and CXB in mice injected with GEM-CXB NPs were prolonged by approximately 3.6 folds and 3.9 folds, respectively. In addition, the area under the plasma drug concentration-time curve (AUC_0‑∞_) increased approximately 2.4-fold for GEM and 2.6-fold for CXB. Furthermore, the clearance (CL) and volume of distribution (Vd) values were obviously lower than those with individual GEM and CXB treatment. The superior pharmacokinetic performance of GEM-CXB NPs enhances the potential of both GEM and CXB for efficient accumulation in tumors.

HPLC was used to determine the distribution of CXB and GEM in different tissues of tumor mice after intravenous injection of CXB solution, GEM solution, and gem-CXB NPs for 24 h. As shown in Fig. 5C and D, the distribution of CXB in the liver and kidney after treatment with CXB free solution was greater than that in the GEM-CXB NPs group (Fig. 5D). This difference may be attributed to the fact that most of the free drugs entered the body and were metabolized in the liver, and then excreted through the kidneys, resulting in lower levels in tumor tissues. Compared with the CXB solution, the content of drugs distributed in tumors increased 2–3 times after GEM-CXB NPs treatment. Simultaneously, the distribution of drugs in the liver and kidney was weakened to some extent and the bioavailability of the drugs improved. This finding provides a basis for the drugs to exhibit a synergistic anti-tumor effect while reducing pathological damage to the liver and kidney.

Meanwhile, the results of tissue distribution show that most of the drugs are distributed in the liver and lung tissues after administration of the GEM free solution (Fig. 5C). This suggests that GEM causes damage to the liver and lung, which is consistent with numerous clinical studies indicating liver and lung toxicity associated with GEM. In contrast, after GEM-CXB NPs treatment, the accumulation of drugs in the liver was reduced, thereby minimizing the damage caused by GEM to these organs. Additionally, there was a significant increase in the amount of drugs distributed to tumor tissues.

The pharmacokinetic study in Fig. 5A and B, as well as Tables S1 and S2, reveals that compared to the single free solution group, GEM-CXB NPs exhibit higher drug accumulation in tumor tissues. This is attributed to the prolonged circulation time of CXB and GEM in the blood when administered as GEM-CXB NPs. Consequently, a higher blood drug concentration is maintained for a longer duration, allowing more GEM-CXB NPs to accumulate in tumor tissues via blood circulation. This improves the distribution of drugs specifically in tumor tissues and reduces toxicity to other tissues. The effective tumor accumulation of GEM in combination with a large number of CXB distributed in tumors achieves the goal of chemotherapy-immunity synergistic tumor eradication.

### In vivo anti-tumor and anti-metastasis

**Fig. 6:**
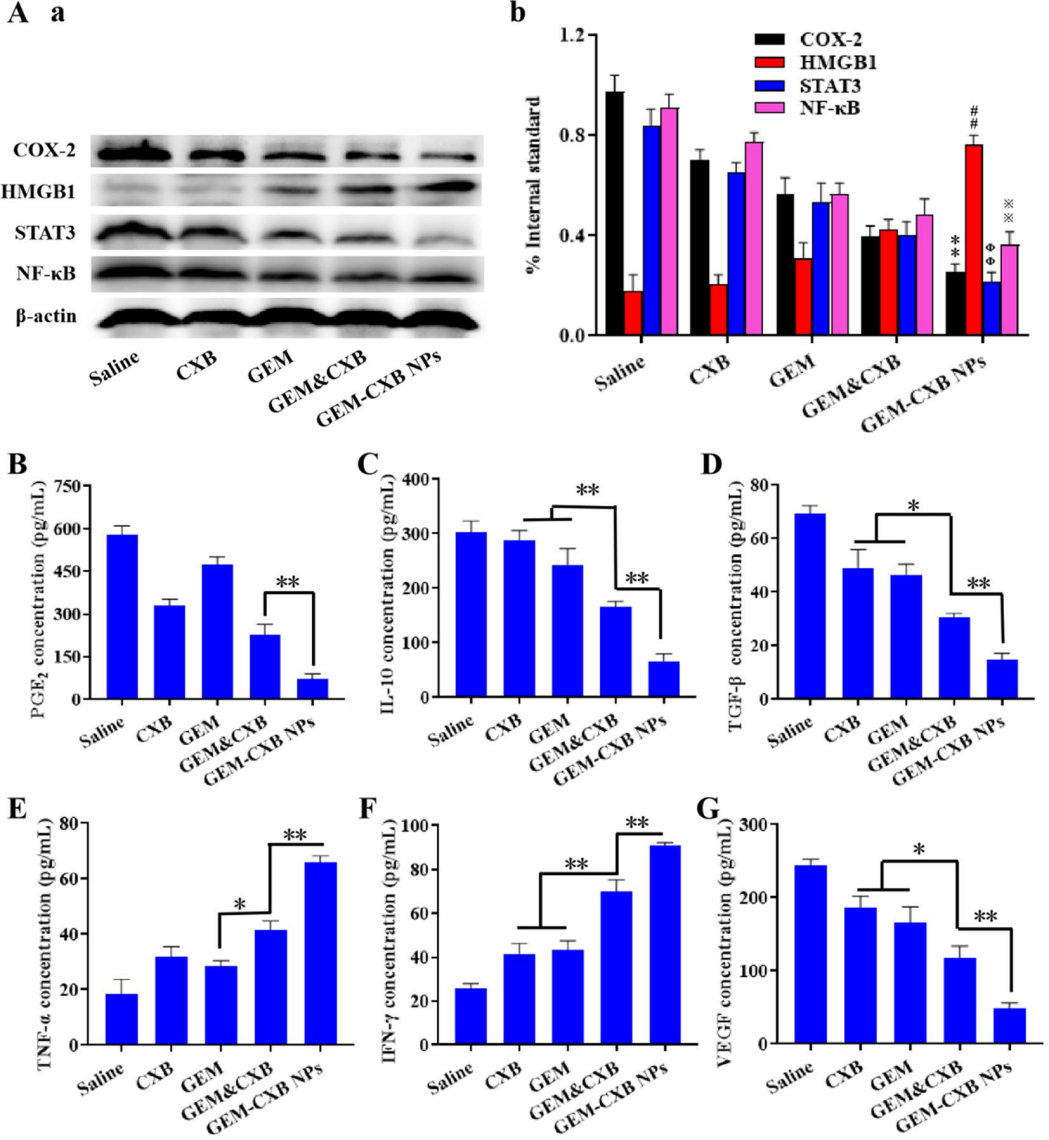
(**A**) Inhibition curve of tumor volume growth, (**B**) body weight changes, (**C**) tumors photographs and (**D**) survival profiles of mice following various formulations-treatment ( ^*^p < 0.05, ^**^p < 0.01, *n* = 6). (**E**) H&E and TUNEL analyses of mice following various formulations-treatment. The scale bar is set to 20 μm. (**F**) Images and quantification of metastatic tumor nodules, and H&E staining (**G**) of the lungs in mice following various formulations-treatment ( ^*^p < 0.05, ^**^p < 0.01, *n* = 6). The scale bar is set to 50 μm

The in vivo antitumor efficacy of GEM-CXB NPs was studied in the BALB/c mice bearing 4T1 tumors. As shown in Fig. 6A and C, and 6D, the saline-treated mice exhibited rapid tumor growth and a short median survival time of only 21 days. Mice administered free CXB showed the least tumor growth inhibition, with a median survival time of 24 days, whereas mice treated with free GEM showed moderate tumor growth inhibition and a longer median survival time of 30 days. Treatment with the GEM&CXB combo led to more effective tumor growth inhibition and prolonged median survival time of 36 days, compared to treatment with GEM or CXB alone, which caused by the synergistic chemo-immune antitumor actions of the combination treatment. Importantly, the best overall antitumor efficacy was observed in mice treated with GEM-CXB NPs, these mice exhibited the strongest inhibition of tumor growth and the most obvious improvement in survival rate, with a mean survival time of 45 days. Histological examination using TUNEL analysis and H&E staining was carried out to further assess the degree of tumor necrosis and apoptosis induced by different treatments (Fig. 6E). The extent of necrosis and apoptosis observed in the tumors correlated with the antitumor effects observed in the study.

The inhibition of lung metastasis by GEM-CXB NPs was studied by examining the number of metastatic nodules in the whole lung (Fig. 6F) and through histological analysis using H&E staining (Fig. 6G). The largest area of lung metastases was observed in the pathological sections and the most number of lung metastatic nodules (19 lesions) was counted in the saline-treated mice. This confirmed the highly metastatic nature of the 4T1 tumors, which readily formed metastatic nodules in the lungs. Treatment with CXB or GEM alone provided slight relief from lung metastases, while the GEM&CXB combo demonstrated a greater anti-metastasis effect on the 4T1 tumor-bearing mice. GEM-CXB NPs showed the highest anti-metastasis activity, with only two lung metastatic nodules, and physiological characteristics almost similar to those of a healthy lung.

Altogether, GEM-CXB NPs demonstrated a remarkable ability to inhibit lung metastasis in BALB/c mice bearing 4T1 tumors, likely attributable to their prolonged blood circulation and preferential accumulation and co-release of GEM and CXB in tumors, contributing to the dual depletion of MDSCs and tumor cells, and consequent generating chemotherapeutic and immune antitumor effects.

### In vivo safety evaluation

The effects of different treatment formulations on mice were assessed in terms of body weight, hematological parameters, and histopathological characteristics of major organs. Figure 6B shows that mice with indicated treatments exhibited steady growth in body weight throughout the monitoring period, indicating good tolerance to the treatments. As shown in Figure S4B-I, compared with other treatment groups, the serum biochemical markers of liver function such as AST and ALT in mice treated with GEM and GEM&CXB alone increased significantly, although there was no significant difference in platelet (PLT) count, the white blood cell (WBC) count decreased significantly. Furthermore, the central veins in the livers of mice with GEM and GEM&CXB combo treatments were dilated and exhibited inflammatory cell infiltration, which was consistent with the clinically reported hepatotoxic activity of GEM in vivo, which can cause mild liver injury. However, mice treated with GEM-CXB NPs showed normal levels of serum biochemical markers, including AST and ALT, as well as normal blood routine indexes of WBC and PLT. Moreover, no histopathological lesions or abnormities were found in the normal organs of these mice, which might be attributed to the decreased liver accumulation of GEM in these mice, results in decreased hepatotoxicity (Figure S4A). These results indicated that GEM-CXB NPs exhibit good in vivo biosafety and could be used as a prospective nano-twin drug delivery system for the chemoimmunotherapy of breast cancer.

### Chemoimmunotherapy mechanisms of the GEM-CXB nano-twin drug

**Fig. 7:**
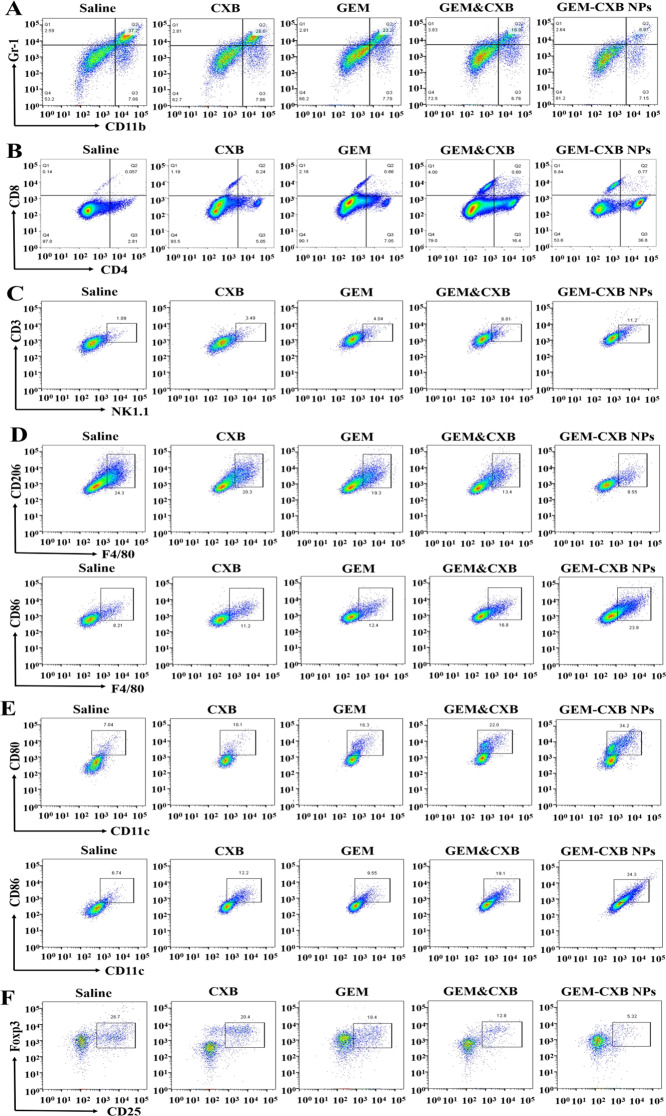
(**A**) The expressions of COX-2, HMGB1, STAT3 and NF-κB in different formulations treated-4T1 tumors determined by western blot (a) and quantified via Image J software (b). ^**^p < 0.01, ^##^*p* < 0.01, ^ΦΦ^*p* < 0.01, ^※※^*p* < 0.01 (GEM-CXB NPs vs. Saline or GEM&CXB). The levels of PGE_2_ (**B**), IL-10 (**C**), TGF-β (**D**), TNF-α (**E**), IFN-γ (**F**) and VEGF (**G**) in different formulations treated-4T1 tumors ( ^*^p < 0.05, ^**^p < 0.01, *n* = 3)

The ITM was jointly regulated by tumor cells, MDSCs, and other immunosuppressive cells through the interaction of multiple signaling pathways to exacerbate cancer progression. The COX-2/PGE_2_ pathway played a key role in tumor immune escape through hindering the migration of matured DCs and recruiting immunosuppressive Tregs and MDSCs [21, 22]. Moreover, the tumor growth and the recruitment and immunosuppression of MDSCs were promoted by NF-κB and STAT3 [9, 13]. Therefore, the chemoimmunotherapy mechanisms underlying the in vivo antitumor and anti-metastasis efficacies of GEM-CXB NPs were explored through ELISA and western blot investigation. As illustrated in Fig. 7a and b, GEM-treated 4T1-tumors had no inhibitory effect on COX-2/PGE_2_ pathway, whereas an increased expression of HMGB1 was observed. On the contrary, CXB treatment had no influence on HMGB1 expression, but exhibited decreased COX-2/PGE_2_ levels in 4T1-tumors. Notably, treatment with GEM&CXB combo led to decreased COX-2/PGE_2_ levels and increased expression of HMGB1, an indicator of ICD. More important, treatment with GEM-CXB NPs exhibited the most effective synergistic actions in suppressing the COX-2/PGE_2_ pathway and activating ICD induction in vivo. In addition, both the CXB and GEM downregulated the expressions of STAT3 and NF-κB, which was attributed to the selective depletion of MDSCs by these two drugs. The GEM&CXB combo treatment displayed synergistic inhibitory activity on STAT3 and NF-κB. Importantly, GEM-CXB NPs showed the lowest expression levels of NF-κB and STAT3, indicating the advantage of co-depletion of MDSCs provided by GEM-CXB NPs. Overall, the combinatory therapy of GEM-CXB NPs, with blockade of the COX-2/PGE_2_ pathway, activation of ICD, and depletion of MDSCs, was found to help activate the antitumor immune response via jointly improving the maturation of DCs, promoting the presentation of TAAs and facilitating to re-modulate the ITM.

**Fig. 8:**
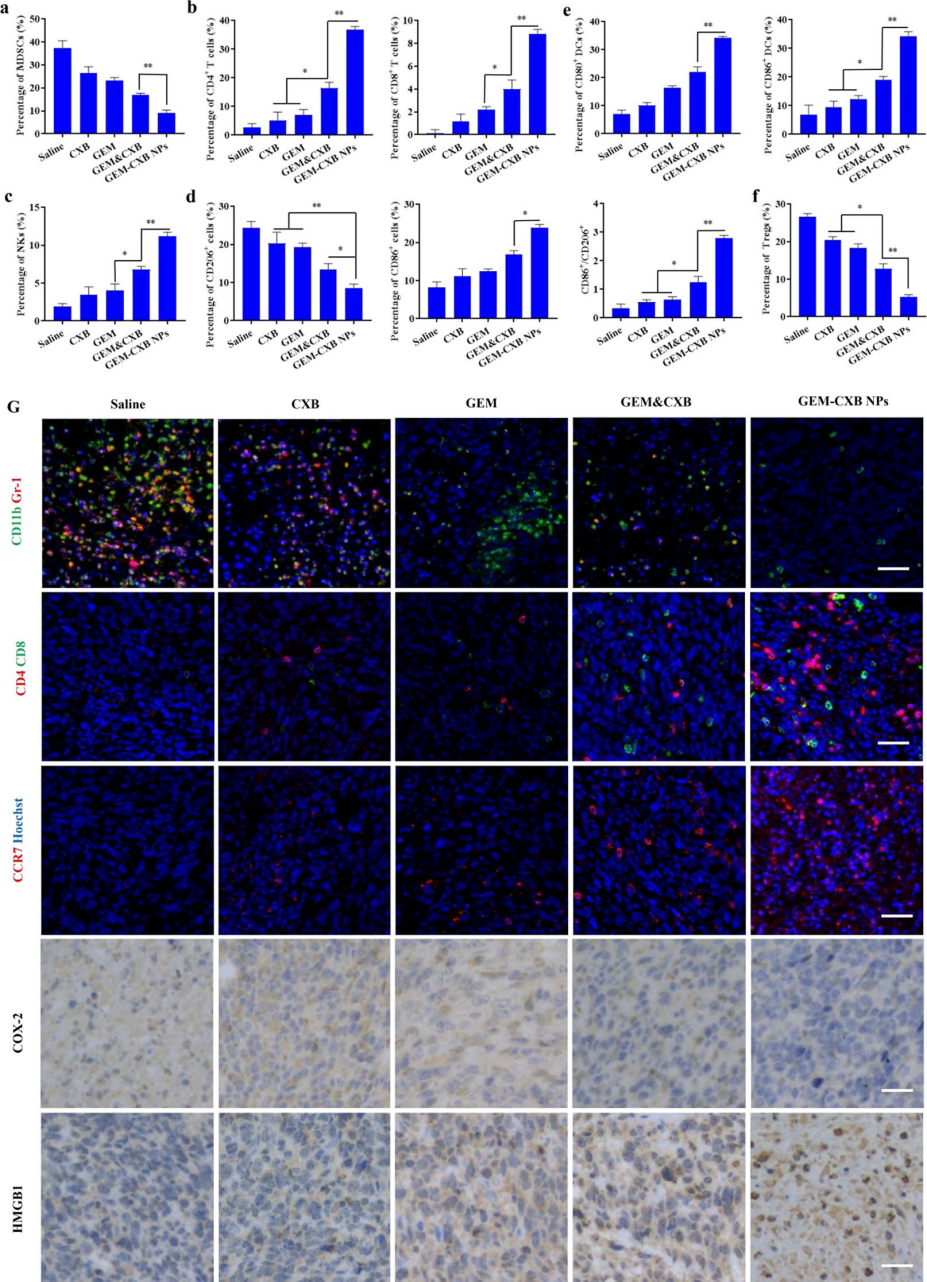
The percentage of CD11b^+^Gr-1^+^ MDSCs (**A**, a), CD11c^+^CD80^+^ DCs, CD11c^+^CD86^+^ DCs (**E**, e), CD4^+^CD8^+^ T cells (**B**, b), NK1.1^+^ (**C**, c), CD25^+^Foxp3^+^ Tregs (**F**, f), F4/80^+^CD206^+^ TAMs and F4/80^+^CD86^+^ TAMs (**D**, d) in different formulations treated-4T1 tumors. Data were expressed as mean ± SD (*p < 0.05, **p < 0.01, *n* = 3). (**G**) Immunofluorescence staining analysis of MDSCs, CD4^+^CD8^+^ T cells and CCR7, and the immunohistochemistry analysis of COX-2 and HMGB1 expressions in different formulations treated-4T1 tumors. The scale bar is 100 μm

The mechanisms underlying the activation of immune responses by GEM-CXB NPs combinatory therapy in 4T1 tumors were performed by flow cytometry. Compared to the control group, free CXB and GEM exhibited depletion activity against CD11b^+^Gr-1^+^ MDSCs, with a stronger effect observed in the GEM&CXB combo (Fig. 8A). Importantly, treatment with GEM-CXB NPs led to the highest suppression of MDSCs, indicating effective alleviation of immunosuppression of MDSCs in the TME. This facilitated the activation of antitumor immune responses by promoting the maturation of DCs, increasing the infiltration of CTLs and NKs, and reducing the proliferation of Tregs. As showed in Fig. 8E, GEM-CXB NPs-treated tumors presented the highest proportions of CD11c^+^CD86^+^ DCs (34.3%) and CD11c^+^CD80^+^ DCs (34.2%), which were 5.1-fold and 4.5-fold higher, respectively, than that in saline-treated tumors. The improved maturation and recruitment of DCs in response to GEM-CXB NPs may be attributed to the synergistic alleviation of immune suppression through the inhibition of the COX-2/PGE_2_ pathway, depletion of MDSCs, and enhanced the induction of ICD, which are conducive to promote antigen presentation and activate the immune response of positive immunomodulatory cells. Therefore, the tumor infiltrations of the CD4^+^ and CD8^+^ T cells in CXB-GEM NPs-treated tumors was analyzed to be approximately 7.0 folds, 4.0 folds and 2.3 folds more than those in CXB, GEM and CXB&GEM combo-treated tumors, respectively (Fig. 8B). Furthermore, the GEM-CXB NPs-treated tumors presented the highest proportion of NKs, re-polarization of TAMs with increased M1/M2 ratios, and the lowest proportions of CD25^+^Foxp3^+^ Tregs (Fig. 8C, D and F).

The secretion of inflammatory cytokines in tumors with indicated treatments was analyzed by ELISA to further examine the activation of antitumor immune response. Depletion of MDSCs resulted in an obvious reduction in IL-10 (Fig. 7C) and TGF-β (Fig. 7D), two major immunosuppressive factors affecting the immunosuppressive activity of MDSCs, in tumors treated with GEM-CXB NPs compared to those receiving other treatments, resulting in increased infiltration of CTLs and decreased recruitment of Tregs in the tumors. Moreover, the levels of TNF-α (Fig. 7E) and IFN-γ (Fig. 7F) secreted in the GEM-CXB NPs-treated tumors were 3.7-fold and 3.6-fold higher, respectively, than that in the saline-treated tumors. These findings indicate a favorable alleviation of immunosuppression in the TME and activation of the antitumor immune response via the dual depletion of tumor cells and MDSCs. Moreover, GEM-CXB NPs-treated tumors exhibited the lowest levels of VEGF (Fig. 7G), which is important for tumor vascular normalization and inhibition of tumor metastasis.

Immunofluorescence and immunohistochemistry analyses were employed to further illustrate the underlying chemoimmunotherapy mechanisms of GEM-CXB NPs (Fig. 8G). Consistent with the results of western bolt and flow cytometry analyses, tumors treated with GEM-CXB NPs exhibited the highest potency in inducing ICD, evidenced by the most effective promotion of HMGB1 release and inhibition of the COX-2/PGE_2_ pathway, while also depleting MDSCs, indicated by the decreased frequency of CD11b^+^Gr-1^+^ MDSCs in tumors. This contributed to a considerable increase in the frequency of CCR7^+^ cells and the highest frequency of CD4^+^ and CD8^+^ T cells in tumors, which indicated effective remodulation of the ITM by GEM-CXB NPs.

In conclusion, the GEM-CXB nano-twin drug combinatory therapy strategy showed significant potential in activating antitumor immune response by eliciting the ICD induction, stimulating the maturation and recruitment of DCs, as well as increasing the infiltration of NKs and CTLs, while simultaneously reducing the infiltration of immunosuppressive cells. Thus, this therapy shows efficacy in suppressing the growth of 4T1 tumors, inhibiting lung metastasis, and prolonging overall survival rates, demonstrating a potent chemoimmunotherapy effect.

## Conclusions

We successfully developed a simplified GEM-CXB nano-twin drug for dual depletion of MDSCs and tumor cells for breast cancer chemoimmunotherapy. The GEM-CXB NPs exhibited prolonged circulation in the bloodstream, preferential accumulation in tumors, and co-release of GEM and CXB in tumors. These in vivo characteristics of GEM-CXB NPs afford an efficient chemotherapeutic activity against 4T1 tumor cells while also activating the antitumor immune response by synergistically depleting MDSCs, thus ultimately achieving enhanced antitumor and anti-metastasis efficacy in mice bearing 4T1 tumors. This GEM-CXB nano-twin drug system, which achieves dual depletion of MDSCs and tumor cells, serves as an innovative strategy for cancer chemoimmunotherapy.

### Electronic supplementary material

Below is the link to the electronic supplementary material.

Supplementary Material 1

## Data Availability

No datasets were generated or analysed during the current study.
