# Supplementary material for: Dual depletion of myeloid-derived suppressor cells and tumor cells with self-assembled gemcitabine-celecoxib nano-twin drug for cancer chemoimmunotherapy
Source: J Nanobiotechnology. 2024 Jun 8;22:319. doi: 10.1186/s12951-024-02598-y (PMC11161946; doi:10.1186/s12951-024-02598-y)
Supplement: Supplementary file 1 — Supplementary Material 1 [file 12951_2024_2598_MOESM1_ESM.docx]

**Supporting information**

**Title:**

**Dual depletion of myeloid**-**derived suppressor cells and tumor cells with self-assembled gemcitabine**-**celecoxib nano**-**twin drug for cancer chemoimmunotherapy**

**Authors’ informations**

**Author:**

Xiaojie Zhang ^1, †^, Qiangwei Liang ^1, 2, †^, Yongjin Cao ^3^, Ting Yang ^1^, Min An ^1^, Zihan Liu ^1^, Jiayu Yang ^1^,Yanhua Liu ^1, *^

**Affiliation:**

^1^Department of Pharmaceutics, School of Pharmacy, Ningxia Medical University, Yinchuan 750004, China

^2^NHC Key Laboratory of Metabolic Cardiovascular Diseases Research, Ningxia Medical University, Yinchuan 750004, China

^3^Department of Pharmacy, School of Nursing, Wuxi Taihu University, Wuxi 214064, China

**Author Contributions:**

^†^ Xiaojie Zhang and Qiangwei Liang contributed equally to this study.

* **Correspondence:**

Yanhua Liu

[lyanhua1214@126.com](mailto:lyanhua1214@126.com)

**Table S1** Main pharmacokinetic parameters of GEM in mice treated with different formulations (mean ± SD, *n* = 6).

| Parameters | GEM | GEM-CXB NPs |
| --- | --- | --- |
| t_1/2β_ (h) | 1.32 ± 0.43 | 4.86 ± 0.66** |
| AUC_0-∞_ (ng^*^h/mL) | 13254.08 ± 539.28 | 32389.28 ± 237.08** |
| V_d_ (L/kg) | 3.51 ± 1.25 | 0.96 ± 0.51** |
| CL (L/h/kg) | 0.23 ± 0.09 | 0.06 ± 0.03** |

**p* <0.05, ***p* <0.01 (GEM-CXB NPs vs GEM)

**Table S2** Main pharmacokinetic parameters of CXB in mice treated with different formulations (mean ± SD, *n* = 6).

| Parameters | CXB | GEM-CXB NPs |
| --- | --- | --- |
| t_1/2β_ (h) | 1.05 ± 0.26 | 4.12 ± 0.37** |
| AUC_0-∞_ (ng*h/mL) | 19514.38 ± 624.73 | 51141.59 ± 826.39** |
| V_d_ (L/kg) | 2.69 ± 0.96 | 0.81± 0.94** |
| CL (L/h/kg) | 0.19 ± 0.15 | 0.07 ± 0.02** |

**p* <0.05, ***p* <0.01 (GEM-CXB NPs vs CXB)


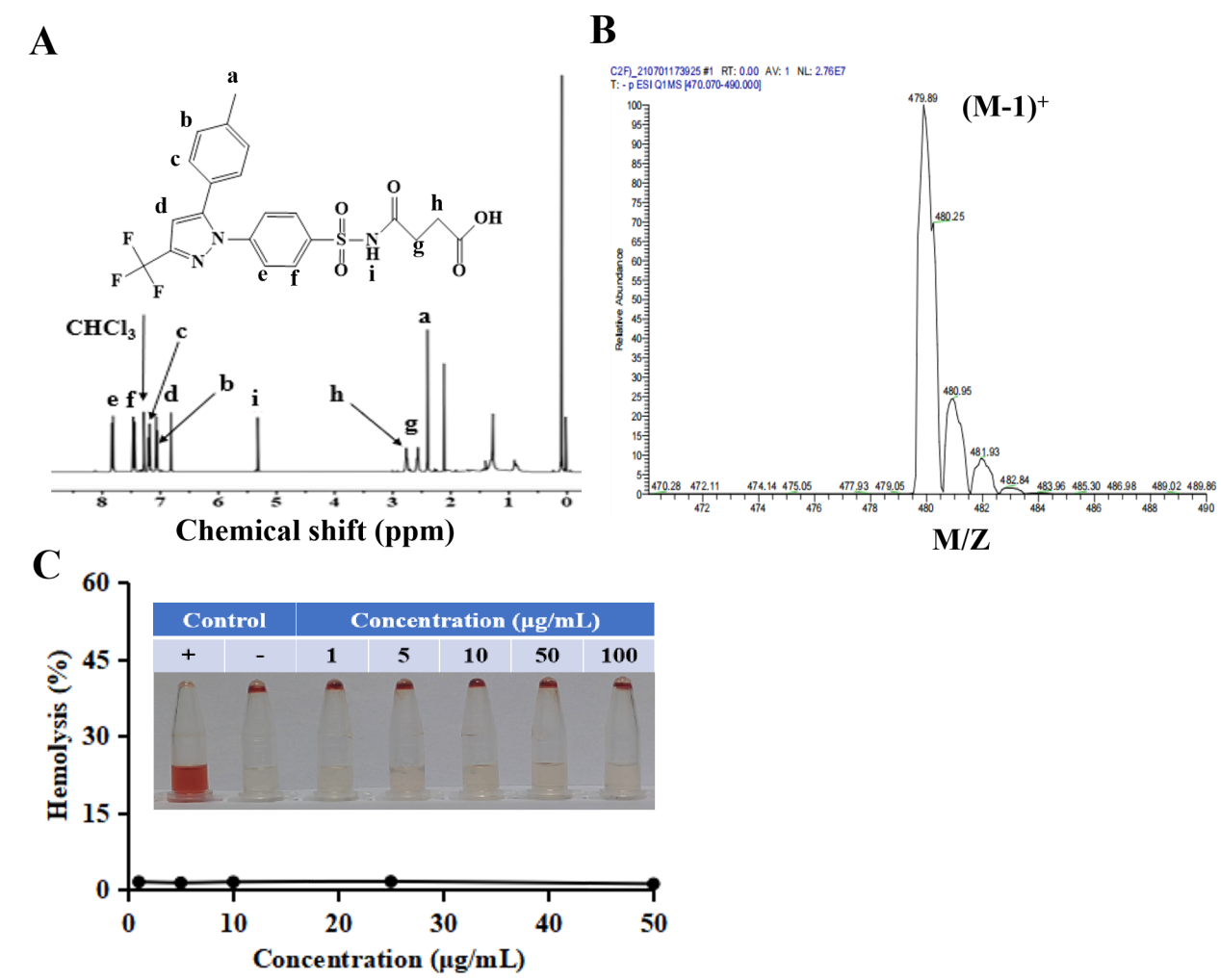


**Figure S1.** (A) ^1^H NMR spectrum of CXB-SA in CDCl_3_ and (B) ESI-MS spectrum of CXB-SA. (C) Hemolysis profile of mice blood treated with GEM-CXB NPs.


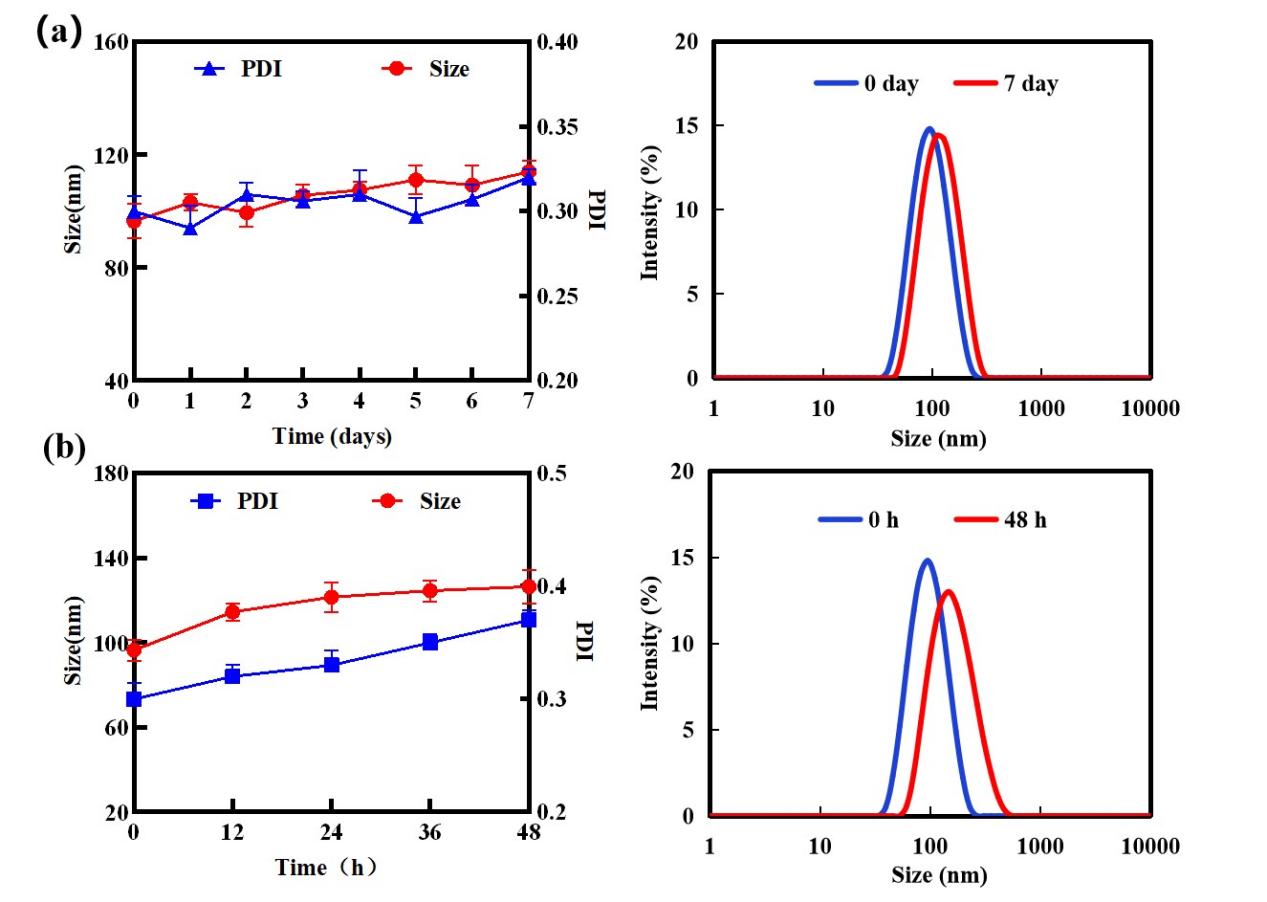

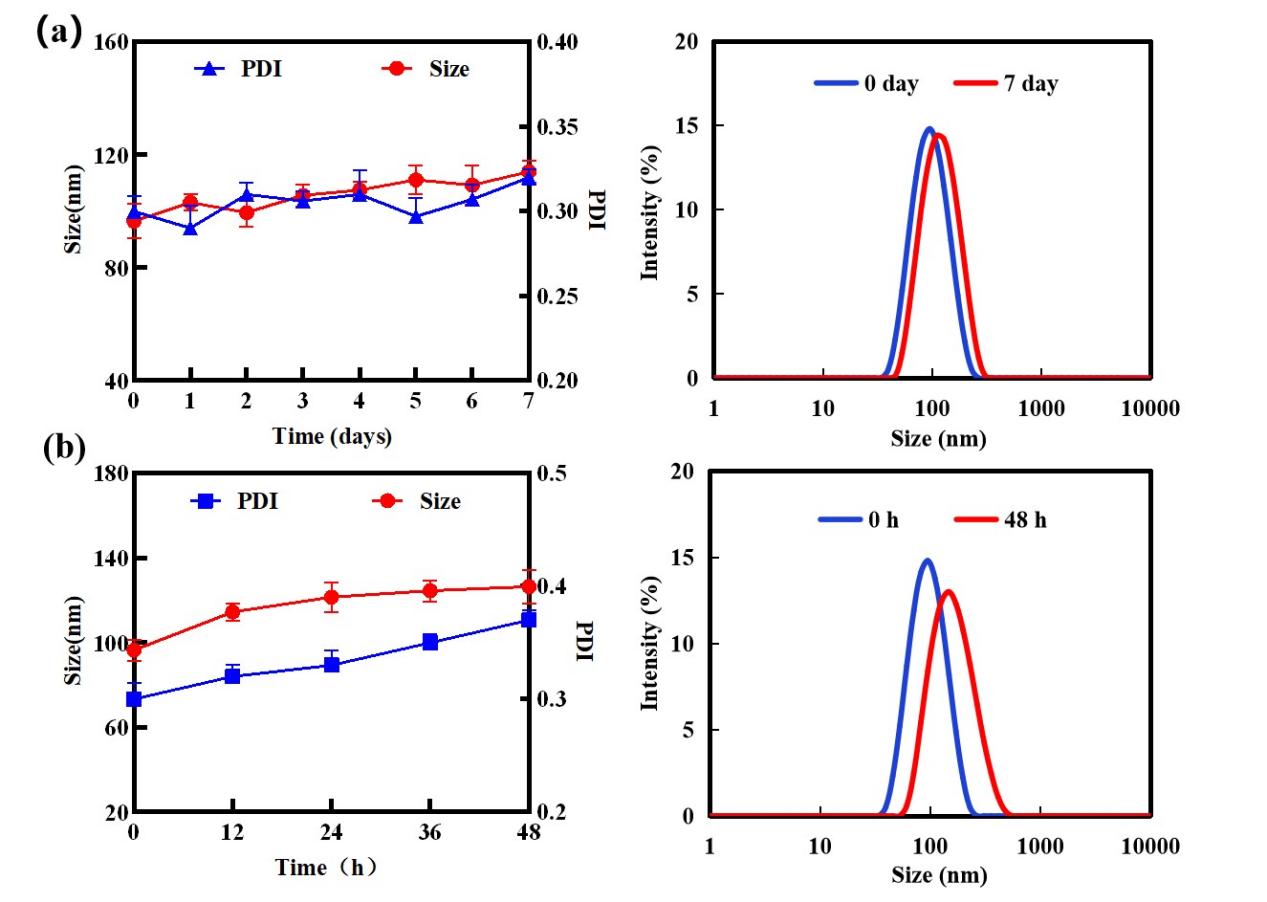


**A**

**B**

**Figure S2.** Particle size and PDI changes of GEM-CXB NPs in pH 7.4 PBS at 4°C (A) and pH 7.4 PBS with 10% FBS at 37°C (B) as the incubation time prolonged.


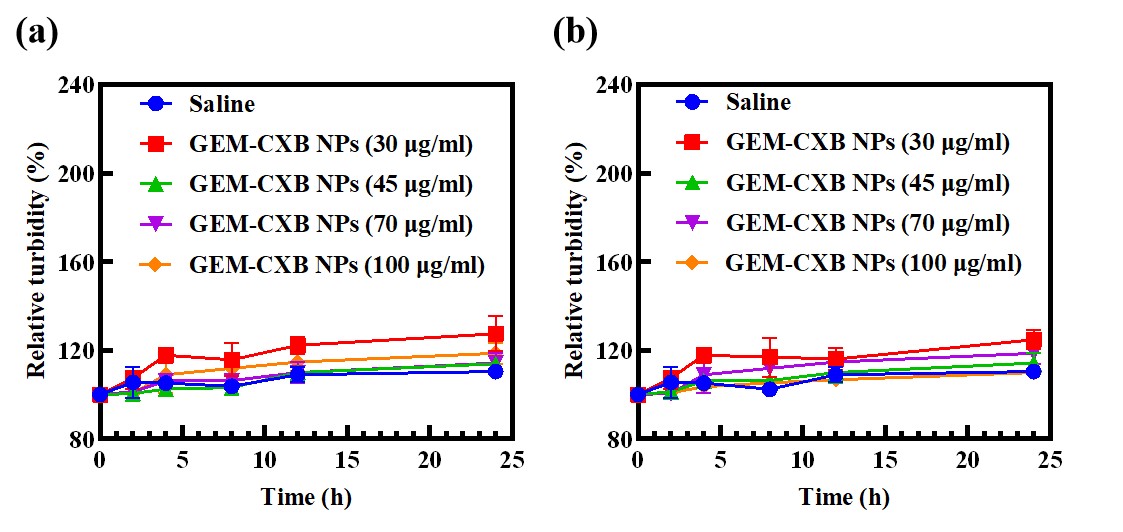


**A**

**B**

**Figure S3.** Effects of GEM-CXB NPs on preventing aggregation induced by plasma (A) and whole blood (B, *n* = 6).


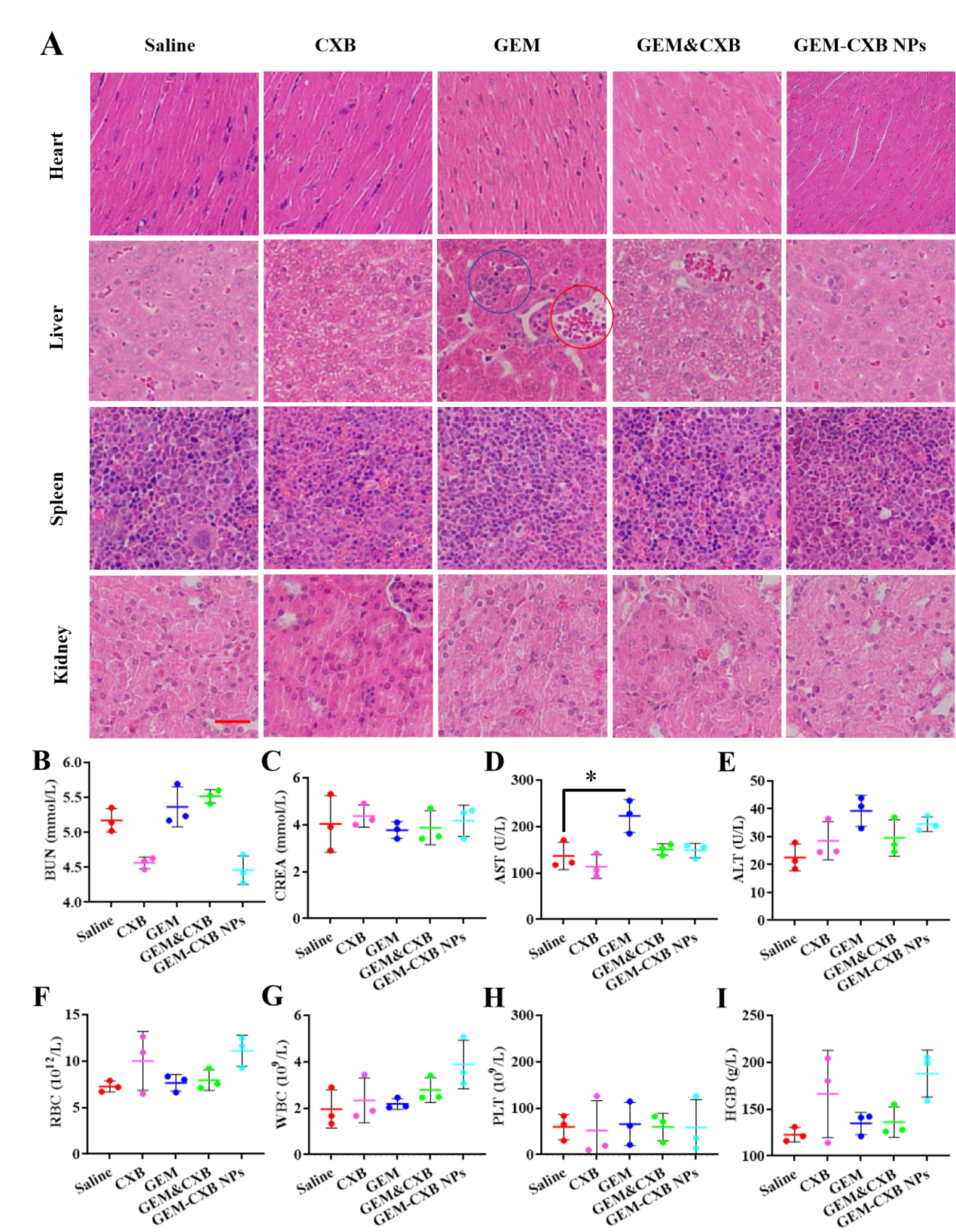


**Figure S4.** (A) H&E staining of major organs of 4T1-tumor mice after different treatments. The scale bar is set to 50 μm. Hemanalysis of BUN (B), CREA (C), AST (D) and ALT (E) levels, and blood biochemical indices of red blood cell (RBC, F), white blood cell (WBC, G), platelet (PLT, H) and haemoglobin (HGB, I) in 4T1-tumor mice with different treatments. ( **p* <0.05, ***p* <0.01, *n* = 3).
